# Rare Complication of Orthognathic Surgery: Intrusion of Mandibular Condyle into the Middle Cranial Fossa

**DOI:** 10.29252/wjps.10.3.111

**Published:** 2021-09

**Authors:** Gholamreza Motazedian, Ali Khojasteh, Fatemeh Salari

**Affiliations:** 1 Department of Plastic and Reconstructive Surgery, Shiraz University of Medical Sciences, Shiraz, Iran; 2 School of Dentistry, Shiraz University of Medical Sciences, Shiraz, Iran

**Keywords:** Mandibular condyle, Middle cranial fossa, Orthognathic surgery

## Abstract

Dislocation of mandibular condyle into the middle cranial fossa is rare but diagnosis and treatment timely is very important due to significant complications. In this paper, we present a very rare case of asymptomatic intrusion of the mandibular condyle into the middle cranial fossa after orthognathic surgery in a 23 year old man from Iran.

## INTRODUCTION

Intrusion of the mandibular condyle into the middle cranial fossa is a rare complication of mandibular trauma^1^^-^^4^. The most common cause is traffic accident ^5^. According to our knowledge, this complication is not reported yet after orthognathic surgery. 

Clinical examination, computerized tomography (CT)-scan and magnetic resonance imaging (MRI) can be useful for diagnosis^2^^, ^^4^.

In this paper we present a case of asymptomatic intrusion of mandibular condyle into the middle cranial fossa after orthognathic surgery discovered accidentally during follow up of patient.

## CASE REPORT

We present a 23 yr old man with chief complaint of facial asymmetry. He had past medical history of left hemifacial lymphatic malformation that underwent surgery in childhood. During surgery buccal branch of left facial nerve was injured. After surgery gradually patient developed dentofacial deformity. After puberty patient underwent prolonged orthodontic treatment (pre and post-op), bimaxillary surgery, genioplasty, mandibular body and angle implants. 

**Figure 1:**
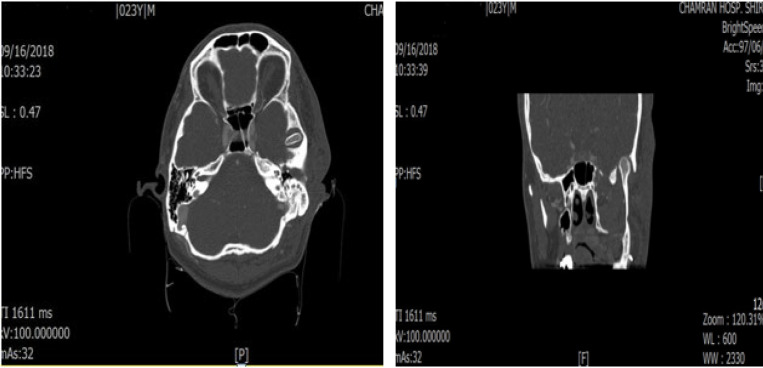
Axial and coronal view of computed tomography images show intrusion of mandibular condyle into middle cranial fossa

In clinical examination his facial countur was not optimal and there was no mouth opening limitation and his occlusion was acceptable with slight deviation of the mandible to the left. Totally patient was asymptomatic. We requested maxillofacial CT-scan. CT-scan revealed intrusion of left mandibular condyle into middle cranial fossa (Figure 1). Because patient was asymptomatic we did not do any surgical Intervention and we decided to follow him regularly.

Informed consent was obtained from the patient. (Ethical Code: IR.SUMS.REC.1400.403).

## DISCUSSION

Mandibular condyle is located inside a cavity within the temporal bone called glenoid fossa. The anterior wall of the glenoid fossa is built by the articular eminence of squamous part of temporal bone and its posterior wall built by the tympanic plate of temporal bone which also forms the anterior wall of external acoustic meatus. The roof of the glenoid fossa is a thin and translucent bone. The anterior and posterior walls of this fossa are well developed compared to its roof ^2^^, ^^6^^-^^8^. Intrusion of mandibular condyle in to middle cranial fossa is a rare form of condylar dislocation especially as a complication of orthognathic surgery. The most common cause of this injury is traffic accident^5^^, ^^9^. One of the factors that increases the risk of this complication is the shape and morphology of the glenoid fossa and head of mandibular condyle. Because the middle part of the roof of the glenoid fossa is thinner than the lateral parts, a small and round head of mandibular condyle can penetrate into middle cranial fossa more easily than the normal scroll-shaped condyle^1^^, ^^7^. Other predisposing factors for this type of dislocation include increased pneumatization of the temporal bone, lack of posterior occlusion or open mouth position when the impact occurs and the presence of a defect in the roof of the glenoid fossa structure^2^^-^^4^^, ^^10^. 

In our case in fact we believe that there may have been a defect in the structure of the glenoid fossa that was related to the left hemifacial lymphatic malformation which weakened the glenoid fossa. 

There are several clinical findings that help for diagnosis of this type of condylar dislocation such as: restriction of mandibular mobility, unstable occlusion, deviation of the jaw toward the side of injury, posterior open bite on the opposite side of injury, cerebrospinal leakage or external auditory canal hemorrhage, facial asymmetry, reduce height of ramus on the side of injury and empty glenoid fossa in examination of temporomandibular joint^1^^, ^^3^^, ^^4^^,^^ 6^^, ^^10^. Because these symptoms are non-specific and can be seen in other mandibular condyle fracture we need to use other diagnostic tools such as CT-scan and MRI^2^^, ^^4^. However, the inferior part of the temporal lobe is a relatively silent area of the brain and epilepsy and other cerebral deficits are uncommon in this region but patient should be evaluated for neurosurgical complications such as cerebrospinal fluid leakage, extradural hematoma and meningitis because the roof of the glenoid fossa is floor of the cranial base and placement of the middle meningeal artery in the floor of the middle cranial fossa in close proximity of the penetration^3^^, ^^4^^, ^^11^.

In our case, the patient complained of facial asymmetry. At the time of referral there was no history of limitation in the mandibular mobility or trauma to the mandible. His occlusion was acceptable and he had no neurologic symptoms. After clinical and radiographic evaluation we concluded that the main reason for the facial asymmetry was the intrusion of the mandibular condyle into the middle cranial fossa although previous buccal nerve injury caused slight asymmetry. 

In the similar studies, different treatments have been recommended according to the existing conditions such as osteotomy of the mandibular condyle and remaining of the condylar head in the middle cranial fossa, condylectomy through the craniotomy, closed reduction and open reduction with removal of the condylar head as well as reconstruction of the glenoid fossa^1^^, ^^3^^, ^^9^. We informed patient his main problem and after talking with him and his parents, we did not do any surgical intervention. We decided to follow him regularly because many years had passed since his orthognathic surgery and he was asymptomatic. 

At the time of writing this paper, the patient had been followed for 2 years. At recent follow up appointment his maximum mouth opening was 40 mm with slight deviation of the mandible to the left. 

To our knowledge this is the first case of the asymptomatic intrusion of the mandibular condyle into the middle cranial fossa as a complication of orthognathic surgery that is reported.

## CONCLUSION

Although the prevalence of intrusion of mandibular condyle into middle cranial fossa as one of the complications of orthognathic surgery is very rare but it should be considered .

## CONFLICT OF INTEREST

The authors declare no conflict of interest.
